# Better Inputs, Better Learning: A Peptide Embedding Tutorial for Proteomic Mass Spectrometry

**DOI:** 10.1021/acs.jproteome.5c00563

**Published:** 2026-01-13

**Authors:** Luke Squires, Jose Humberto Giraldez Chavez, Alfred Nilsson, Lukas Käll, Samuel H Payne

**Affiliations:** 1 Biology Department, 6756 Brigham Young University, Provo, Utah 84602, United States; 2 Science for Life Laboratory, School of Engineering Sciences in Chemistry, Biotechnology and Health, KTH Royal Institute of Technology, Stockholm 17165, Sweden

**Keywords:** machine learning, proteomics AI, tutorials, proteomics education, peptide, embedding, encoding

## Abstract

Mass spectrometry proteomics creates complex data representing the peptide/protein contents of biological samples. Various types of machine learning have been central to computational methods used to identify peptides from tandem mass spectra and numerous other aspects of the data analysis process. As deep learning has emerged as a powerful machine learning method for modeling and interpreting data, computational proteomics researchers have leveraged large publicly available data sets to train machine learning models to predict peptide fragmentation spectra and liquid chromatography retention time. Resources like proteomicsML offer extensive demonstrative tutorials for these learning tasks and are closing the gap between the proteomics and machine learning communities. However, in these and other educational materials on deep learning, the critical step of preparing data for learning is frequently omitted. Prior to learning, peptide strings must be converted into a numeric format—an embedding. There are many different peptide embeddings, and some vastly outperform others. Yet the process for creating an embedding, and also the rationale for choosing a specific embedding, is rarely discussed in our proteomics literature. In this technical note, we introduce four Google Colab notebooks to teach peptide embeddings. The series walks users through five different peptide-embedding strategies— from simplistic single-number encodings to state-of-the-art pretrained embeddings— through both code examples and narrative descriptions. The final notebook compares the five embeddings in a head-to-head benchmark. By making these notebooks free, we hope to lower the barrier for researchers who want to bring modern deep learning into their proteomics workflows.

## Graphical abstract

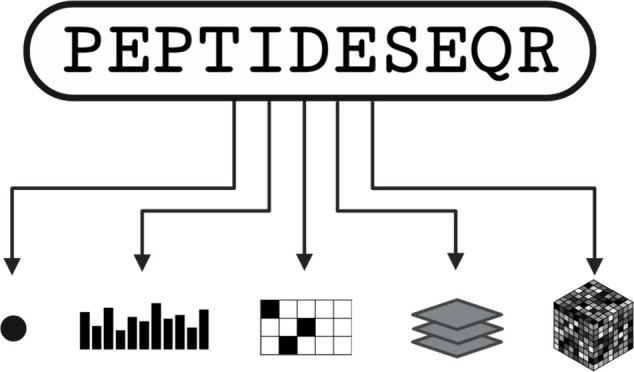

## Introduction

Machine learning is a driving force in many aspects of mass spectrometry proteomics data analysis algorithms and software,^1^ with early work often focused on scoring functions and methods to improve MS2 identifications.^2–4^ As the past decade has seen a massive increase in publicly available proteomics data and coincident increase in computational power, machine learning (ML) has become increasingly important in many areas within our data analysis pipelines. Although current aspirations as a community may extend toward the simulation of an entire proteomics experiment,^5^ we are far from the ability to predict most aspects of the proteomics data generation process. For predictive ML, a few key areas of success are predicting MS/MS fragmentation, peptide retention time, and the peptides most likely to be observed after tryptic digestion.^6–11^

Unlike the large domains of natural language processing and image processing, where ML has generalized beyond a small group of academic researchers, machine learning for proteomics still remains a niche domain. Advancing ML research within proteomics will require outreach to those beyond computational mass spectrometrists. A growing global interest in machine learning and its transformative potential in science has helped juxtapose two potentially disparate audiences.^12^ Among the current proteomics practitioners, there are many with training in biology, chemistry and physics who demonstrate an interest in crossing over into computational proteomics. A second audience are those in computer science, applied math and data science who are looking for a motivating scientific domain. We see an opportunity to bring these two groups together and bridge the technical aspects of machine learning with the scientific goals of proteomics and its unique data types. Current resources like proteomicsML.org demonstrate numerous learning tasks and help to classify the types of questions that can be asked with mass spectrometry data.^13^ Most of the ML tasks start with peptide sequences as a primary input. Yet these resources lack an explanation of how peptide sequences are transformed and prepared for input to ML.

To use peptides as input to a machine learning tool, the sequence of amino acids must first be converted to a numeric representation. A peptide sequence is natively text, but ML is a mathematical optimization problem that works exclusively with numeric data. The representation of data in its (often lower-dimensionality) numeric form is called an *embedding*. Peptide embeddings have evolved significantly, with the representations growing increasingly complex. Some early efforts in proteomics machine learning used just a single number as the representation of a peptide, e.g., the retention coefficient.^14^ Other embeddings enumerate the composition of amino acids.^8,15,16^ A more advanced and feature-rich representation often includes some notion of the chemical properties of the amino acids, e.g., size, charge, hydrophobicity, beta-sheet forming propensity, etc. These features are hand-crafted, derived from a researcher’s expert knowledge. The number and type of hand-crafted features differs significantly between tools, ranging from a few dozen^17,18^ to hundreds of features.^6^

The current wave of machine learning centers on deep neural networks, sometimes called deep learning. A significant advance within deep learning is moving away from hand-crafted embeddings and to a machine-learned representation. Much of today’s research focuses on pretraining large models to capture the structure of specific data types. Just as GPT models have revolutionized natural language processing^19^ and DINO has transformed image understanding,^20^ similar efforts are underway in proteomics. Instead of manually engineering features to represent peptides, we can now train large models to learn rich, high-dimensional embeddings of amino acid sequences^21–23^ or mass spectra.^24^ These pretrained models can then be fine-tuned or used as components in larger generative frameworks, enabling powerful transfer learning across predictive tasks in proteomics.

In this technical note, we announce a series of four computational tutorials that are designed to explore peptide embedding methods. Peptides are the input to many of the common ML applications, e.g. predicting retention time, predicting MS/MS fragmentation, predicting proteotypic peptides, etc. As the input in any learning task, individuals wanting to do proteomics machine learning need to understand embeddings — both philosophically and also technically. Our goal with the tutorials is to bridge the gap between machine learning and proteomics, reaching out to machine learning practitioners and proteomics researchers alike and help them understand how to start.

## Methods

We use Google Colab as a platform for creating a series of four tutorials. These interactive Python notebooks can integrate rich mark-down text and python code. Our tutorial notebooks are open source and freely available. Please see the links at https://github.com/PayneLab/ProteomicsEducation.

### Tutorial 1: Simple Embeddings

Tutorial 1 covers three methods for creating embeddings, which are all intuitive and require minimal coding. The three methods are single number embeddings using pI (isoelectric point), fractional amino acid composition and one-hot encoding.

#### Method 1 – Isoelectric Point (pI)

Although the isoelectric point (pI) is not typically used as an embedding feature in practice, we chose it as a starting point to help users understand how abstract biological concepts—like a peptide—can be represented numerically through real-world properties. In Method 1, we use an existing python package to calculate the pI for a given sequence.

#### Method 2 – Fractional Amino Acid Composition

We next introduced the idea of representing a peptide using multiple numbers through the fractional composition method.^15^ Because the source code used in the original manuscript was not available, we recreated and adapted it from the description in their methods section. Unlike a single-value embedding used in Method 1, this method uses an array that contains the relative amount of a given amino acid inside of a peptide, with each position in the array representing the proportion of that amino acid to everything else.

#### Method 3 – One-Hot Encoding

One-hot encoding is a common machine learning transformation and is frequently used in proteomic research.^16,25–27^ One-hot encoding creates an n-by-m matrix, where n is the length of the peptide and m is 20 (the number of amino acids). For each position 0 < i < n, the i-th amino acid is marked in the matrix as being present with a 1, and all other values in that column are marked as zero. This embedding preserves amino acid order as well as composition.

### Tutorial 2: Complex Embeddings

Tutorial 2 covers only one method for making a complex embedding - the atomic embedding introduced by Bouwmeester et al.^28^

#### Method 4 – Atomic Composition and PTM Encoding

Like previous methods, Method 4 is based on a published approach.^28^ This method expands on previous work in several important ways. First, it highlights the shortcomings of the one-hot encoding and its inability to work with post-translational modifications. Second, it expands the embedding space beyond a matrix to a set of matrices (a tensor).

### Tutorial 3: Learned Embeddings

Tutorial 3 covers only one method for getting a complex embedding - the ESM model, an open-source protein language model.^23^

#### Method 5 – Learned Embeddings with Deep Learning

In Method 5, we discuss learned embeddings which are generated by deep learning models trained on large protein sequence data sets. All prior embeddings were created via a hand-crafted set of features. However, a learned embedding is derived via the deep learning process. Therefore, the meaning of values in the tensor is not dictated by research and impossible to explain. The tutorial uses the ESM (Evolutionary Scale Modeling) model and demonstrates how to use the ESM code to transform their peptide sequence into a multidimensional tensor embedding.

### Tutorial 4: Benchmarking Embeddings

Using data from proteomicsML, we benchmark the success of learning to predict peptide retention time using the input of each of the 5 methods of peptide embeddings. We obtained 7383 preformatted peptides and a retention time from their GitHub repository - https://github.com/ProteomicsML/ProteomicsML/blob/main/datasets/retentiontime/PXD028248/PXD028248_evidence_selected_columns.zip?raw=true. These are used with a standard neural network learning regime.

After creating embeddings using the methods above, we flatten all embeddings into one-dimensional (1D) arrays to make sure that each method feeds into the same neural network architecture. This was to make sure that the only difference between embedding methods is in how the sequence is represented, not the model architecture. We then paired the embedding with their corresponding RT and fed them into a PyTorch-based regression model. We split our data set into training, validation, and test sets: train: ∼ 68% of the total data, validation: ∼ 12% of the total data, test: 20% of the total data. All code for the model architecture, training and evaluation can be found in the tutorial code.

## Results

Using Google’s interactive Python Colab notebooks (https://colab.research.google.com/), we created a four part tutorial for peptide embeddings as they are used in proteomic machine learning. Five different methods for creating an embedding are shown: single numeric value, list of amino acid composition, one-hot encoding, atomic composition, and a learned embedding from ESM (Box [Boxed-text box1] and Methods). As an educational tool, an important feature of these tutorials is the description of the goals and purposes of each different type of embedding. Interspersed throughout the notebooks are reflective questions, prompting the reader to consider how they might accomplish a specific task or whether there are shortcomings to a presented method. For example, we ask readers to think about how two similar peptide sequences might be represented by an embedding, e.g. would the embedding capture important similarities and differences? In addition to these text-based materials, the tutorials demonstrate the technical coding used to create each embedding.

1 **Growing complexity of peptide embeddings that represent the peptide for machine learning. Alternative methods for embedding peptide information**

1. A single numerical value (scalar). Two examples are the isoelectric point, or the retention coefficient.

2. A one-dimensional list of values (array or vector). One implementation is the fractional composition of amino acids in the peptide.

3. A two-dimensional grid of values (matrix). The most common matrix representation is a one-hot encoding showing amino acid position within the peptide.

4. A three-dimensional block of hand crafted features (tensor). Shown here is the atomic encoding from ref,^28^ which contains a series of matrices.

5. A three-dimensional block with learned features (tensor). For this embedding, the values are entirely derived from machine learning. They are a useful but often difficult to interpret representation.

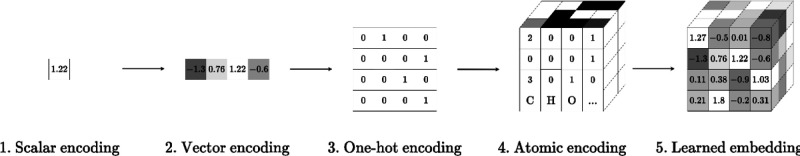

Two essential topics for discussion about machine learning inputs (embeddings) is what information is provided and how that information is derived. As can be seen in Box [Boxed-text box1], the increasing complexity of embeddings corresponds to more information being handed to the machine learning process. With method #1, only a single number is given. Thus, the math transformations in the training and learning process have a very limited set of information to work with. Methods #2, 3, and 4 move from vectors to matrices to tensors. In this progression, more information about the input is added, providing the training process a better opportunity to understand the inputs and transform them into accurate outputs.

The second topic, how information is derived, is an important shift in machine learning in recent years. Note that in Box [Boxed-text box1], the first four options all provide information that is handcrafted by the ML engineer. However, with #5 the embedding is itself the product of ML training. Although it may have started with a set of simple handcrafted features, this embedding is the result of many rounds of training and optimization. In the case of the ESM model described in tutorial 3, this training utilized millions of protein sequences and structures to learn a meaningful representation of amino acid sequences.

The final tutorial is a benchmarking of the five different peptide embeddings against a simple machine learning task. Due to its ease of demonstration and widespread application in proteomics, we choose to benchmark against predicting a peptide’s liquid chromatography retention time. The tutorial downloads the data, produces peptide embeddings for all five methods, and then trains a modest neural network. Regardless of what type of embedding is used, the architecture of the network remains the same. The goal of this benchmarking is to demonstrate whether there is a performance advantage due to the embeddings.

We emphasize that predicting retention time is not the only machine learning task that utilizes peptide embeddings. Rather, it is used simply as a concrete and relatable example. Moreover, we acknowledge that our retention time training uses a modest size data set. Most high performance models of retention time prediction use millions of peptides. This amount of training data also requires a normalized retention time across dozens of LC-MS runs which often use different gradient lengths, e.g. 15, 60, 100 min, etc. Therefore, for simplicity in this tutorial example and to help keep the focus on embeddings as the primary topic, we use a small scale data set for benchmarking.

To evaluate the performance of each embedding, we compared the training and validation loss after each epoch (Figure 1). The most simplistic representations (methods 1 and 2) have only modest performance. A significant difference between these embeddings and those derived from methods 3, 4, and 5 is representing amino acid order within the peptide. Indeed the embeddings from methods 2 and 3 really only differ in that the one-hot encoding captures the sequence ordering in addition to the composition. This subtle difference, however, results in a significant difference in performance.

**Figure 1:**
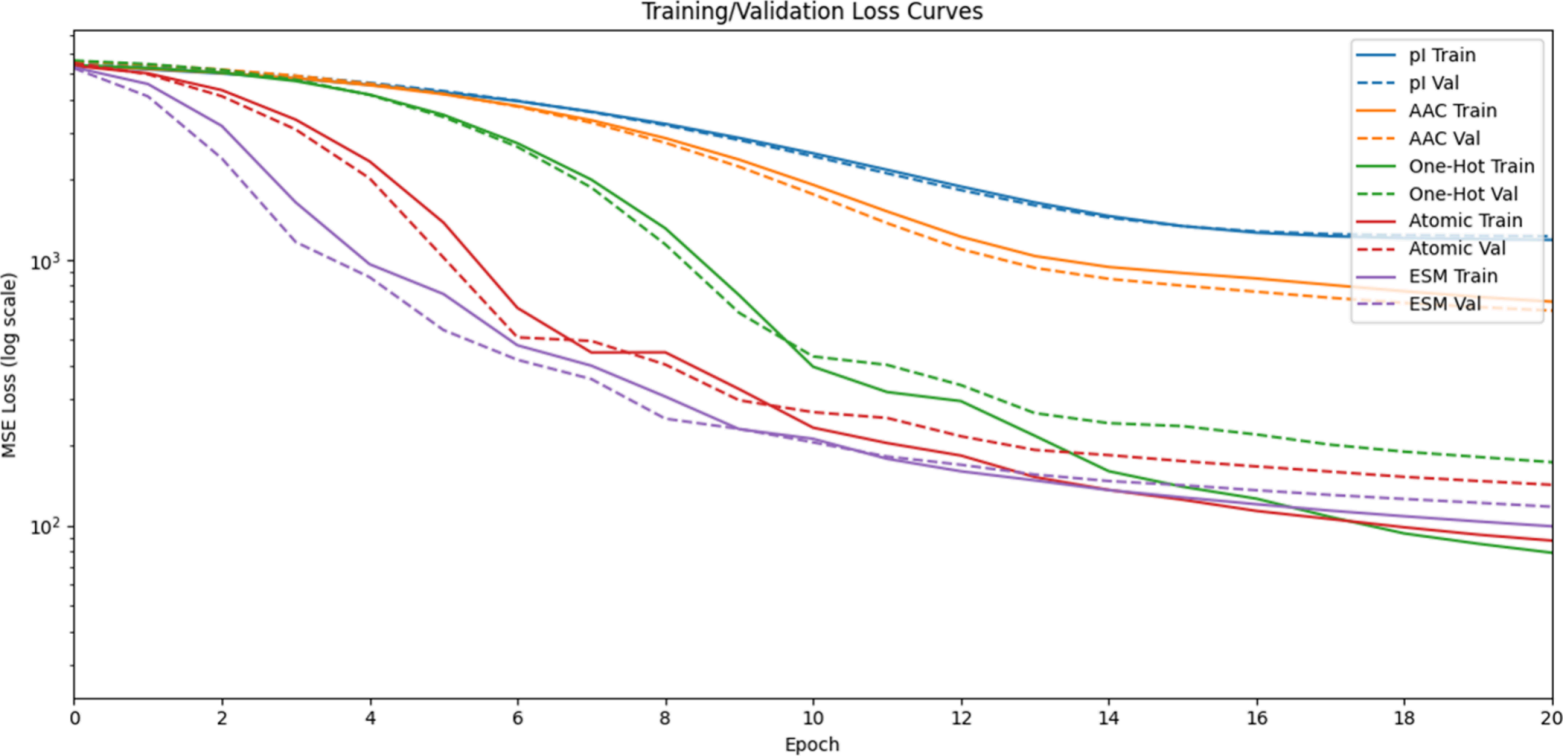
Model loss during training. After each epoch of training, we measure model accuracy through the loss function on both training and validation data. Smaller loss values equate to a more accurate model. We note that for visual clarity, we plot here only 20 epochs. However, the model continued to learn and decrease error. A full training of the model would extend for more than 50 epochs before validation loss failed to decrease.

In evaluating the performance of a machine learning model, it is essential to separate the performance on peptides from the training and validation data sets. As is standard practice, a random sample of peptides are periodically chosen from the training and validation set to see how accurately the model predicts the retention time. The peptides within the training set are directly used for model creation. Thus, the model has learned from these exact peptides beforehand. However, peptides from the validation set are separated from training and their errors are not used in fitting the model. This distinction is critical for understanding when a model has finished learning. In Figure 1, note that the solid and dashed lines diverge in later rounds of training (e.g., around epoch 13 for the green data representing the one-hot encoding). After this point, the error for training data continues to significantly improve, while the error measured for the validation set decreases at a slower rate. This indicates that the model has become overfit to the training data. When looking for the best model, we only compare the results on peptides from the validation set. Thus, the ESM model (purple dashed line in Figure 1) has the best performance.

Determining when a model has finished learning requires an evaluation of the marginal gain in performance. For the purposes of this benchmarking, we stopped training after 50 epochs when most models had converged or were producing limited marginal improvement. After 50 epochs, we finally evaluate each model’s performance on the test data - peptides that have been held out and never used by the model during training. As shown in Figure 2 and Table 1, the model trained with ESM embeddings has the best performance, although embeddings from methods 3, 4, and 5 are all reasonable. We again note that these models were created with a limited amount of data and are for demonstration purposes. Training with a larger data set (e.g., millions of peptides instead of thousands) will substantially improve the accuracy of prediction.

**Figure 2:**
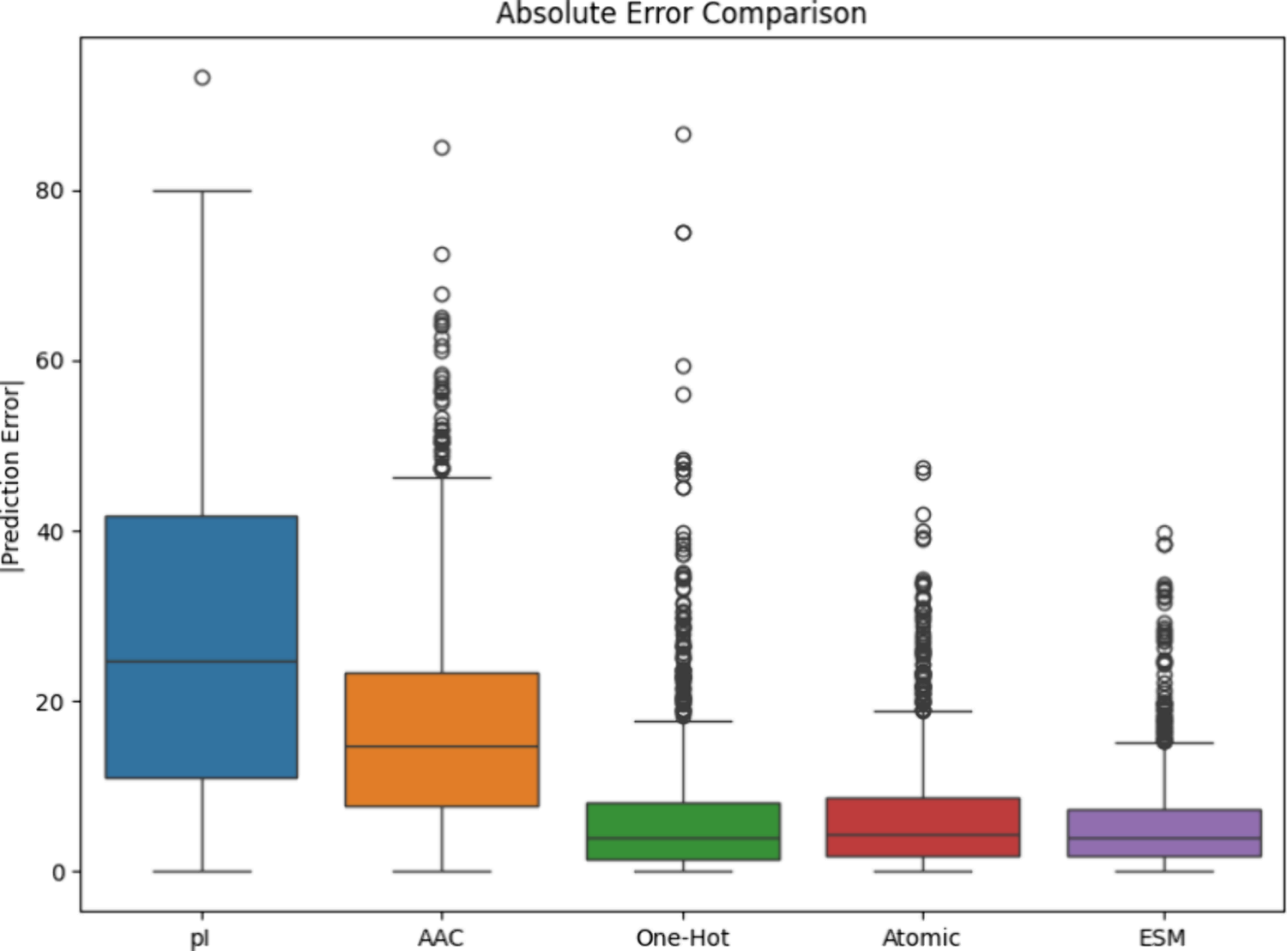
Retention time prediction error of fully trained models. Error is calculated by the difference between observed and predicted retention time for the 1477 peptides in the test data set. The model trained with ESM peptide embeddings has the best performance, with the lowest median error and smallest interquartile range.

**Table 1:** Model Progress during Training^a^

	after 10 epochs			after 20 epochs			after 50 epochs		
	Abs error	test *R* ^2^	test residual IQR	Abs error	test *R* ^2^	test residual IQR	Abs error	test R^2^	test residual IQR
pI	40.48	–1.08	51.17	27.83	0.01	49.09	27.28	0.07	48.92
AAC	36.25	–0.73	52.21	22.14	0.39	39.60	17.00	0.62	29.12
one-hot	15.82	0.64	26.30	8.63	0.88	13.42	6.22	0.91	7.81
atomic	15.07	0.70	24.38	8.80	0.88	13.08	6.50	0.93	8.93
ESM	11.63	0.81	18.34	7.98	0.90	12.62	5.77	0.95	8.27

a During training, we evaluated the model errors. Shown below are snapshots of the median absolute error of the test set, the R2 score (coefficient of determination from regression), and the interquartile range for the absolute error of prediction.

## Discussion

Data transformation prior to machine learning is often overlooked in educational materials, which tend to focus on specific architectures and optimization techniques. Thus, for a domain with non-numeric data types such as proteomics, the learning curve for machine learning can be steep and unclear. Here, we present a series of computational notebooks that explain the philosophical and technical methods for peptide embeddings. Furthermore, we demonstrate that the specific method used to create an embedding has a significant impact on the performance of a machine learning tool. For the benchmarking task shown here, the most successful of these embeddings, those derived from deep learning of foundation models for protein sequences, can be borrowed via APIs and thus require minimal coding to use for new projects. Pretrained embeddings such as ESM are already optimized representations, often capturing biologically meaningful information and enabling better performance with less task-specific training data. The drawback of these advanced embeddings is their size, as they require very large disk space relative to a simple one-hot encoding.

The notebooks that we present here are part of a growing series of educational materials for computational proteomics. Due to the significant interdisciplinary aspects of proteomics (e.g chemistry, molecular biology, computing, statistics, etc.), it is challenging for new individuals to join our field and for current researchers to maintain knowledge and technical skills in applications such as machine learning. We hope that outreach efforts like these will help to increase our reach and recruit others to our field. Future educational materials for computational proteomics could include topics such as spectral embeddings, transfer learning, model fine-tuning, and the train/validate/test cycle for machine learning.

## Data Availability

Our tutorial notebooks are open source and freely available. Please see the links at https://github.com/PayneLab/ProteomicsEducation.

## References

[1] Bouwmeester R., Gabriels R., Van Den Bossche T., Martens L., Degroeve S. (2020). The Age of
Data-Driven Proteomics: How Machine Learning Enables Novel Workflows. Proteomics.

[2] Dancík V., Addona T. A., Clauser K. R., Vath J. E., Pevzner P. A. (1999). De Novo
Peptide Sequencing via Tandem Mass Spectrometry. J. Comput. Biol. J. Comput. Mol. Cell Biol..

[3] Elias J. E., Gibbons F. D., King O. D., Roth F. P., Gygi S. P. (2004). Intensity-Based
Protein Identification by Machine Learning from a Library of Tandem
Mass Spectra. Nat. Biotechnol..

[4] Käll L., Canterbury J. D., Weston J., Noble W. S., MacCoss M. J. (2007). Semi-Supervised
Learning for Peptide Identification from Shotgun Proteomics Datasets. Nat. Methods.

[5] Neely B. A., Dorfer V., Martens L., Bludau I., Bouwmeester R., Degroeve S., Deutsch E. W., Gessulat S., Käll L., Palczynski P., Payne S. H., Rehfeldt T. G., Schmidt T., Schwämmle V., Uszkoreit J., Vizcaíno J. A., Wilhelm M., Palmblad M. (2023). Toward an
Integrated Machine Learning
Model of a Proteomics Experiment. J. Proteome
Res..

[6] Mallick P., Schirle M., Chen S. S., Flory M. R., Lee H., Martin D., Ranish J., Raught B., Schmitt R., Werner T., Kuster B., Aebersold R. (2007). Computational
Prediction of Proteotypic Peptides for Quantitative Proteomics. Nat. Biotechnol..

[7] Liu K., Li S., Wang L., Ye Y., Tang H. (2020). Full-Spectrum Prediction
of Peptides Tandem Mass Spectra Using Deep Neural Network. Anal. Chem..

[8] Gessulat S., Schmidt T., Zolg D. P., Samaras P., Schnatbaum K., Zerweck J., Knaute T., Rechenberger J., Delanghe B., Huhmer A., Reimer U., Ehrlich H.-C., Aiche S., Kuster B., Wilhelm M. (2019). Prosit: Proteome-Wide
Prediction of Peptide Tandem Mass Spectra by Deep Learning. Nat. Methods.

[9] Palmblad M. (2006). Retention
Time Prediction and Protein Identification. Methods Mol. Biol. Clifton NJ..

[10] Moruz L., Käll L. (2017). Peptide Retention Time Prediction. Mass Spectrom. Rev..

[11] Wen B., Zeng W.-F., Liao Y., Shi Z., Savage S. R., Jiang W., Zhang B. (2020). Deep Learning in Proteomics.
Proteomics.

[12] Nitz A. A., Mongane A. R., Squires L., Payne S. H. (2024). Attracting Computational
Researchers to Proteomics. J. Am. Soc. Mass
Spectrom..

[13] Rehfeldt T. G., Gabriels R., Bouwmeester R., Gessulat S., Neely B. A., Palmblad M., Perez-Riverol Y., Schmidt T., Vizcaíno J. A., Deutsch E. W. (2023). ProteomicsML: An Online Platform for Community-Curated
Data Sets and Tutorials for Machine Learning in Proteomics. J. Proteome Res..

[14] Palmblad M., Ramström M., Bailey C. G., McCutchen-Maloney S.
L., Bergquist J., Zeller L. C. (2004). Protein Identification by Liquid
Chromatography-Mass Spectrometry Using Retention Time Prediction. J. Chromatogr. B Analyt. Technol. Biomed. Life. Sci..

[15] Petritis K., Kangas L. J., Ferguson P. L., Anderson G. A., Pasa-Tolić L., Lipton M. S., Auberry K. J., Strittmatter E. F., Shen Y., Zhao R., Smith R. D. (2003). Use of Artificial
Neural Networks for the Accurate Prediction of Peptide Liquid Chromatography
Elution Times in Proteome Analyses. Anal. Chem..

[16] Yang Y., Liu X., Shen C., Lin Y., Yang P., Qiao L. (2020). In Silico
Spectral Libraries by Deep Learning Facilitate Data-Independent Acquisition
Proteomics. Nat. Commun..

[17] Moruz L., Tomazela D., Käll L. (2010). Training,
Selection, and Robust Calibration
of Retention Time Models for Targeted Proteomics. J. Proteome Res..

[18] Webb-Robertson B.-J.
M., Cannon W. R., Oehmen C. S., Shah A. R., Gurumoorthi V., Lipton M. S., Waters K. M. (2010). A Support Vector Machine Model for
the Prediction of Proteotypic Peptides for Accurate Mass and Time
Proteomics. Bioinforma. Oxf. Engl..

[19] Radford, A. ; Narasimhan, K. ; Salimans, T. ; Sutskever, I. Improving Language Understanding by Generative Pre-Training, 2018. https://cdn.openai.com/research-covers/language-unsupervised/language_understanding_paper.pdf.

[20] Oquab, M. ; Darcet, T. ; Moutakanni, T. ; Vo, H. ; Szafraniec, M. ; Khalidov, V. ; Fernandez, P. ; Haziza, D. ; Massa, F. ; El-Nouby, A. ; Assran, M. ; Ballas, N. ; Galuba, W. ; Howes, R. ; Huang, P.-Y. ; Li, S.-W. ; Misra, I. ; Rabbat, M. ; Sharma, V. ; Synnaeve, G. ; Xu, H. ; Jegou, H. ; Mairal, J. ; Labatut, P. ; Joulin, A. ; Bojanowski, P. DINOv2: Learning Robust Visual Features without Supervision. arXiv 2023. 10.48550/ARXIV.2304.07193.

[21] Bepler T., Berger B. (2021). Learning the Protein
Language: Evolution, Structure,
and Function. Cell Syst..

[22] Brandes N., Ofer D., Peleg Y., Rappoport N., Linial M. (2022). ProteinBERT: A Universal Deep-Learning
Model of Protein
Sequence and Function. Bioinforma. Oxf. Engl..

[23] Hayes T., Rao R., Akin H., Sofroniew N. J., Oktay D., Lin Z., Verkuil R., Tran V. Q., Deaton J., Wiggert M., Badkundri R., Shafkat I., Gong J., Derry A., Molina R. S., Thomas N., Khan Y. A., Mishra C., Kim C., Bartie L. J., Nemeth M., Hsu P. D., Sercu T., Candido S., Rives A. (2025). Simulating 500 Million Years of Evolution
with a Language Model. Science.

[24] Sanders, J. ; Yilmaz, M. ; Russell, J. H. ; Bittremieux, W. ; Fondrie, W. E. ; Riley, N. M. ; Oh, S. ; Noble, W. S. Foundation Model for Mass Spectrometry Proteomics. arXiv May 19, 2025. 10.48550/arXiv.2505.10848.

[25] Tiwary S., Levy R., Gutenbrunner P., Salinas Soto F., Palaniappan K. K., Deming L., Berndl M., Brant A., Cimermancic P., Cox J. (2019). High-Quality MS/MS
Spectrum Prediction
for Data-Dependent and Data-Independent Acquisition Data Analysis. Nat. Methods.

[26] Guan S., Moran M. F., Ma B. (2019). Prediction of LC-MS/MS
Properties
of Peptides from Sequence by Deep Learning. Mol. Cell. Proteomics MCP.

[27] Wen B., Li K., Zhang Y., Zhang B. (2020). Cancer Neoantigen Prioritization
through Sensitive and Reliable Proteogenomics Analysis. Nat. Commun..

[28] Bouwmeester R., Gabriels R., Hulstaert N., Martens L., Degroeve S. (2021). DeepLC Can
Predict Retention Times for Peptides That Carry As-yet Unseen Modifications. Nat. Methods.

